# Functional Outcome of Internal Fixation (INFIX) in Anterior Pelvic Ring Fractures

**DOI:** 10.7759/cureus.36134

**Published:** 2023-03-14

**Authors:** Muqtadeer Ansari, Abhay Kawedia, Hari H Chaudhari, Yogesh R Teke

**Affiliations:** 1 Department of Orthopedics, Government Medical College and Hospital, Aurangabad, Aurangabad, IND; 2 Department of Orthopedic Surgery, Jamia Islamia Ishaatul Uloom's (JIIU) Indian Institute of Medical Science and Research, Aurangabad, IND

**Keywords:** traumatic pelvic fractures, unstable pelvic ring injury, minimally invasive pelvic ring fixation, anterior pelvic ring injury, infix

## Abstract

Introduction

Pelvic injuries account for 2% of all orthopedic admissions and are associated with high mortality rates. They need a stable fixation and not an anatomical fixation. Hence, the role of internal fixation (INFIX) comes into play, which provides a stable internal fixation without the complication of open reduction and external fixation with plates and screws.

Materials and methodology

Thirty-one patients with unstable pelvic ring injuries coming to a tertiary care hospital in the state of Maharashtra, India, were selected retrospectively. They were operated on with INFIX. Patients were followed up for a period of six months and evaluated according to the Majeed score.

Results

There was a significant functional outcome in patients operated on with INFIX in pelvic ring injuries in terms of the ability to sit, stand, rejoin work, take part in sexual intercourse, and bear pain. An average Majeed score of 78 with signs of a stable bony union by six months and a full range of motion was noticed in most patients with no problems in day-to-day work.

Conclusion

INFIX provides stable internal fixation of pelvic fractures with good functional outcome without the disadvantages of external fixation or open reduction with plates.

## Introduction

Pelvic fractures are high-velocity fractures most commonly caused due to road traffic accidents, which are now on the rise due to better road infrastructure and faster automobiles all over the developed world. Most of the fractures are associated with injuries to other systems, mainly the urogenital and gastrointestinal systems, and also chest, head, and spinal injuries. The posterior ring structures are responsible for most of the pelvic ring stability, whereas the pubic symphysis that holds the pubis together only accounts for 15% of the stability [1]. These cases must be handled by a trauma team consisting of a general surgeon, an intensivist, and an orthopedic surgeon at a setup with the facilities of an intensive care unit (ICU).

Pelvic injuries have been shown to account for 2% of all orthopedic admissions and 3% of all skeletal injuries [2]. High mortality and morbidity rates of 40%-50% in these fractures are due to accompanying injuries to other systems and hemodynamic instability [3]. Advanced trauma life support (ATLS) protocols will be followed first to stabilize the patient, and then, bony injuries are examined with standard examination protocols. Initial stabilization with a pelvic binder has shown to have a tamponade effect, which helps in reducing blood loss and regaining hemodynamic stability; along with it, both the great toe and the knees are also strapped together. Pelvic ring fracture fixation using external fixators is still considered a mainstay of emergency management as fixation leads to hemostasis [4]. It prevents further disruption of fracture fragments and their associated soft tissue injuries. Internal fixation (INFIX) is an alternative to an external fixator, with the advantages of being biomechanically stronger than an external fixator due to its internal profile, being more comfortable for patients, having no risk of pin tract infections, and having no secondary surgeries [5]. INFIX is a better treatment modality for anterior pelvic ring plating in young females as it does not hamper normal childbirth. The range of motion and mobility are achieved faster in INFIX cases, leading to reduced cases of deep vein thrombosis (DVT) and bed sores seen previously in patients treated with external fixators. Nevertheless, external fixators are the treatment modalities of choice in compound pelvic injury patients. INFIX is not yet considered worldwide as a treatment modality for anterior pelvic ring injuries. Hence, this study was undertaken to review the outcome of INFIX in 31 patients with unstable anterior pelvic ring injuries.

Radiographic evaluation using a single anterior-posterior view of the pelvis along with both hips is sufficient for diagnosing and classifying pelvic ring fractures using the Young and Burgess classification. As the pelvis is a ring structure, a minimum of two injuries will be present when a fracture occurs; so, special X-rays must be done to identify all the fracture segments. Special X-rays with inlet and outlet obturator and iliacus views are required to plan the mode of operative management, which consists of external fixation, INFIX, cortico-cancellous screws, plates, or even conservative management. CT scans are required for exact bony reconstruction and MRI for any soft tissue injuries.

## Materials and methods

Thirty-one patients were selected via the inclusion and exclusion criteria, and their data was accessed retrospectively by reviewing their case files after approval from the institutional ethics committee of Government Medical College, Aurangabad (approval number: NOC/15/2023). The patients presented to a government tertiary care center in Maharashtra, India, with unstable pelvic ring injuries between August 2020 and August 2022. They were operated on with INFIX with polyaxial pedicular screws and subcutaneous spinal rods. Initial stabilization was done, and radiographic evaluation was done once the patient was vitally stable. The fracture pattern was classified according to the Young and Burgess classification.

Initially, patients were stabilized hemodynamically, and injuries to other systems were ruled out. The fracture was managed with pelvic binders, bed rest, and bed sore prevention. Once the initial preoperative workup was done, the operative plan was chalked out after a thorough study of all X-rays and CT scans, the examination of skin condition, and general well-being.

The patients were followed up for six months retrospectively. Follow-up was graded based on functional outcomes using the Majeed score [6]. The Majeed score uses five criteria for functional assessment after pelvis injuries, which are pain, sitting, standing, sexual intercourse, and work. They are graded as excellent (>85), good (70-84), fair (55-69), and poor (<55).

The Inclusion criteria consisted of patients with unstable pelvic ring injuries, closed pelvic ring injuries, pubic symphysis separation of more than 2.5 cm, age of more than 18 years, and patients who gave written consent for surgery.

The exclusion criteria consisted of patients with compound pelvic ring injuries as they are better managed with external fixators, age of less than 18 years, failure or refusal to give written consent for surgery on the part of a patient, and Glasgow Coma Scale (GCS) of less than eight due to other system involvement.

Once the 31 patients were selected (21 males and 10 females), with an average age of 46, they were followed up for a period of six months retrospectively. Subsequently, the following data was recorded from their case files: days of hospital stay, days spent in the ICU if any, operative time, intraoperative blood loss, postoperative complications, and number of weeks for radiological signs of union.

Operative procedure

INFIX or internal fixation is a treatment modality for anterior pelvic ring injuries, which consists of using polyaxial pedicular screws inserted from the anterior inferior iliac spine into the supra-acetabular region of the ilium on both sides.

**Figure 1:**
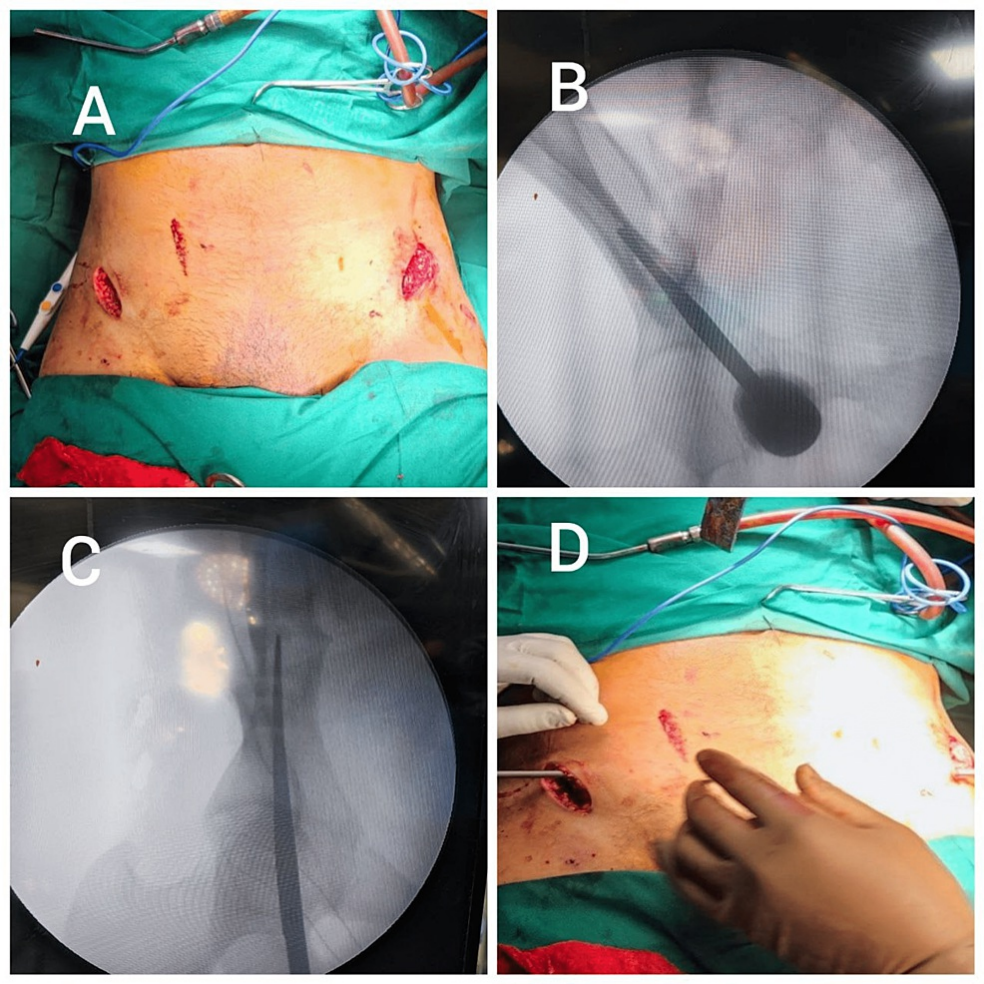
A) Incision taken over the anterior inferior iliac spine. B) Obturator outlet view showing teardrop sign helping guide the entry awl. C) Obturator inlet oblique view or Leeds views showing the correct placement of the awl. D) The subcutaneous placement of the spinal rod.

Essentially, the anterior inferior iliac spine is identified using the teardrop view as a guide [7]; a small incision is then made, and blunt dissection is carried out, creating a plane between the sartorius and tensor fascia lata to reach the anterior inferior iliac spine (Figure 1).

Entry is made with the help of an entry awl, directed toward the supra-acetabular region with the help of c-arm-guided shoots in obturator inlet oblique view (Leeds view) and obturator and iliacus outlet view [8,9].

Polyaxial pedicular screw, usually of size 6.5-8.5 mm in diameter and 80-110 mm in length, is then inserted carefully, allowing it to find its own path. Once the entry is done, the pedicular screw is kept around 20-25 mm above the cortex, maintaining a rod-to-bone distance [10]. The same procedure is then carried out on the opposite side, and the two screws are connected with a subcutaneous spinal rod, which is contoured and sits under an area called the bikini line. The length of the spinal rod is cut short, making sure that there is no irritation to the lateral femoral cutaneous nerve. The rod is compressed, and screws are tightened. The rod-to-symphysis distance was maintained at <40 mm to prevent lateral cutaneous nerve damage [10].

## Results

Thirty-one patients were selected, in which 21 were male and 10 were female, with an average age of 46 years. The main mode of injury was road traffic accidents (23 cases) followed by falls from height (seven cases) and a single case of a pedestrian getting hit by a vehicle. The pelvic fracture was classified according to the Young and Burgess classification shown in Table 1. In 25 patients, posterior fixation was also done along with anterior INFIX. The average number of days spent in the hospital was 16 days (range from 10 to 33 days), 16 patients needed an ICU stay at an average of nine days + 2.5 days (range from six to 13 days), the mean blood loss was 125 ml (range from 40 ml to 350 ml) that when compared to blood loss in open reduction and fixation with plates is much less, and the average time for surgery was 83 minutes (range from 45 minutes to 120 minutes).

**Table 1:** Young and Burgess classification. APC, anterior-posterior compression; LC, lateral compression

Young and Burgess classification	Number of patients
LC type 1	1
LC type 2	7
LC type 3	6
APC type 2	8
APC type 3	9

**Figure 2:**
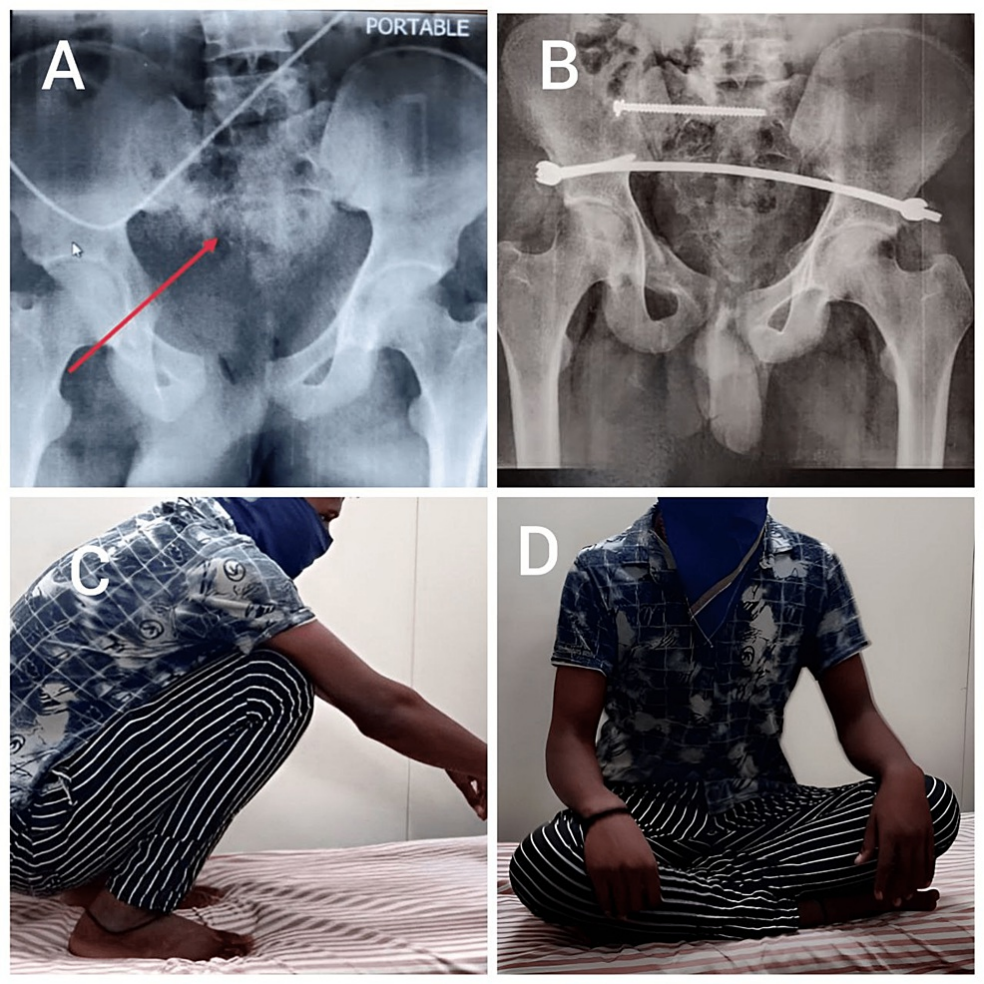
A) A 32-year-old male with anterior and posterior pelvic ring injury. B) Postoperative X-ray of the same patient. C and D) Range of motion at six months.

**Figure 3:**
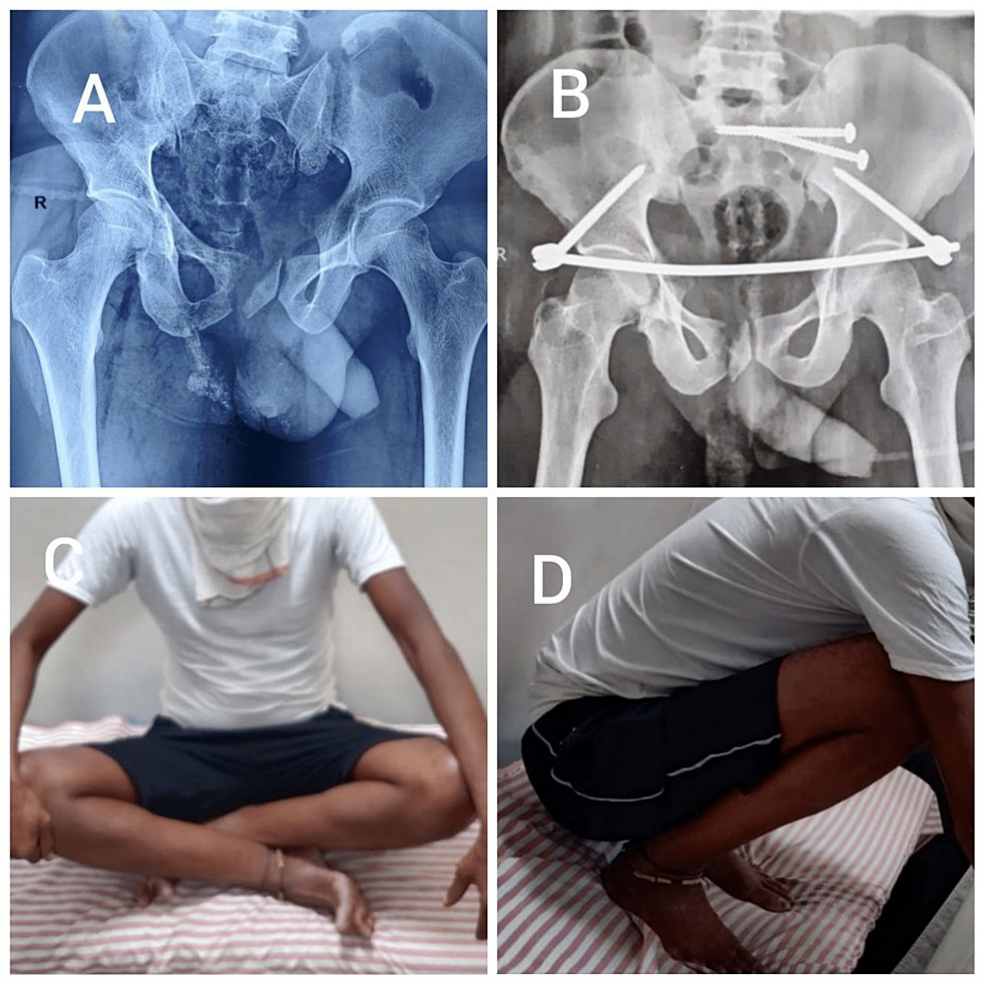
A) A 54-year-old male with anterior and posterior pelvic ring injury. B) Immediate postoperative X-ray. C and D) Range of motion at six months.

The patients were followed up for a period of six months, and regular radiological testing was done, which showed an average time of 14 weeks for union. Three patients showed signs of lateral femoral cutaneous nerve neuralgia and were relieved after implant removal. Four patients showed signs of heterotrophic ossification. One patient showed signs of superficial infection and screw backout, and the implant had to be removed. None of the patients showed signs of deep infection. The Majeed score showed an average of 78 + 12.4 (range of 46-98), with nine patients showing excellent results, 17 showing good results, five showing fair results, and one showing poor result. INFIX was removed in 16 patients only at an average time of 22 weeks (Figures 2-3).

At the time of discharge, all patients were able to sit by the bedside with 90 degrees of flexion at the hip joint and complete range of motion at the knee joint. The patients were mobilized on day 2, and thus, no patients suffered from DVT or bed sores. Regular follow-up was maintained, which showed bony union that was not always anatomical but did not hamper with the range of motion of day-to-day activities of the patient.

## Discussion

Pelvic ring fractures are high-velocity traumas and are primarily associated with road traffic accidents, but low-energy trauma may also lead to pelvic injuries as seen in the elderly population [11]. There are multiple treatment modalities for these patients with pelvic ring injuries, from cortico-cancellous screw fixation to external fixators, open reduction, internal fixation using plates, and a newer modality of internal-external fixation.

Vaidya et al. were of the opinion that INFIX is a viable alternative in case of fractures of the pelvic ring in obese patients, in whom the rods of the external fixator will compress the abdomen [5]. Patients with other system involvement leading to exploratory laparotomy are managed better with plate fixation in the same setting. Internal fixation with plates provides a better reduction of the fracture fragments but at the same time leads to major blood loss, the loss of fracture hematoma, and periosteal stripping. Mishra et al. found that external fixation is the ideal treatment for Tile type B fractures, but it is associated with pin loosening, pin tract infection, being not accepted by patients cosmetically, restricted activity, and osteomyelitis [2]. INFIX provides the same benefits as an external fixator without its disadvantages. Mason et al. conducted a study of 52 patients managed with anterior pelvic external fixators and concluded that 50% of the patients showed pin tract infection [12]. External fixators are still the treatment of choice for grade 3 compound acetabular fractures. There are decreased mortality rates and transfusion needs in these patients when treated with an external fixator [13].

INFIX is a newer modality for treating anterior pelvic ring injuries, with results comparable to external fixators with reduced complications. It is a minimally invasive procedure with small incisions. INFIX is not cumbersome or bulky as compared to an external fixator, providing a better and early range of motion to the patient. It achieves internal fixation without complications such as pin tract infection and pin loosening. No secondary surgeries are required. It is suitable for young female patients of childbearing age as anterior acetabular plates are avoided.

Our study consisted of patients operated on with INFIX over a period of two years at a tertiary care center. The average Majeed score was 78, demonstrating results comparable to that of external fixation and open reduction with internal fixation. It is a simpler, less tedious procedure with minor complications of lateral femoral cutaneous nerve irritation, which according to Scherer et al. can be minimized by maintaining a rod-to-bone distance of 20-25 mm and a rod-to-symphysis distance of less than 40 mm [10]. Femoral nerve palsy is a grave but rare complication of this surgery, and so, meticulous blunt dissection between the sartorius and tensor fascia lata is advised. Screw backout is another complication that warrants implant removal but is rarely seen if screws reach the thick supra-acetabular region.

The study has a few limitations that the author acknowledges. The study has only 31 participants; this subject requires a larger study to accurately depict the functional outcome for pelvic ring injuries operated with INFIX. The study is also a retrospective study that has its limitations. Patients were followed up for a period of six months only; a longer study with a follow-up of at least two years will provide better information about the long-term effects of INFIX.

## Conclusions

Thirty-one patients were operated on with INFIX and followed up for a period of six months retrospectively. The average Majeed score was 78, showing that the functional outcome is favorable and that INFIX can be used as a suitable modality for the definitive management of pelvic ring injuries. It is a safe and easy technique for the management of even complex pelvic ring injuries. It provides a stable fixation giving a satisfactory bony union and adequate functional results even in unstable pelvic ring injuries.

## References

[1] Vrahas M, Hern TC, Diangelo D, Kellam J, Tile M (1995). Ligamentous contributions to pelvic stability. Orthopedics.

[2] Mishra S, Satapathy D, Zion N, Lodh U (2022). Early outcome analysis of management of closed pelvic ring fractures in emergency: conservative versus surgical at level III trauma center in India. Cureus.

[3] Coppola PT, Coppola M (20001). Emergency department evaluation and treatment of pelvic fractures. Emerg Med Clin North Am.

[4] Poka A, Libby EP (1996). Indications and techniques for external fixation of the pelvis. Clin Orthop Relat Res.

[5] Vaidya R, Nasr K, Feria-Arias E, Fisher R, Kajy M, Diebel LN (2016). INFIX/EXFIX: massive open pelvic injuries and review of the literature. Case Rep Orthop.

[6] Majeed SA (1989). Grading the outcome of pelvic fractures. J Bone Joint Surg Br.

[7] Wang MY (2012). Percutaneous iliac screws for minimally invasive spinal deformity surgery. Minim Invasive Surg.

[8] Tosounidis TH, Giannoudis PV (2017). Use of inlet-obturator oblique view (Leeds view) for placement of posterior wall screws in acetabular fracture surgery. J Orthop Trauma.

[9] Guimarães JA, Martin MP 3rd, da Silva FR (2019). The obturator oblique and iliac oblique/outlet views predict most accurately the adequate position of an anterior column acetabular screw. Int Orthop.

[10] Scherer J, Tiziani S, Sprengel K, Pape HC, Osterhoff G (2019). Subcutaneous internal anterior fixation of pelvis fractures-which configuration of the InFix is clinically optimal?-a retrospective study. Int Orthop.

[11] Abdelrahman H, El-Menyar A, Keil H (2020). Patterns, management, and outcomes of traumatic pelvic fracture: insights from a multicenter study. J Orthop Surg Res.

[12] Mason WT, Khan SN, James CL, Chesser TJ, Ward AJ (2005). Complications of temporary and definitive external fixation of pelvic ring injuries. Injury.

[13] Burgess AR, Eastridge BJ, Young JW (1990). Pelvic ring disruptions: effective classification system and treatment protocols. J Trauma.

